# Crawling to Collapse: Ecologically Unsound Ornamental Invertebrate Fisheries

**DOI:** 10.1371/journal.pone.0008413

**Published:** 2009-12-22

**Authors:** Andrew Rhyne, Randi Rotjan, Andrew Bruckner, Michael Tlusty

**Affiliations:** 1 New England Aquarium, Edgerton Research Laboratory, Boston, Massachusetts, United States of America; 2 Roger Williams University, Department of Biology and Marine Biology, Bristol, Rhode Island, United States of America; 3 Harvard University, Department of Organismic and Evolutionary Biology, Cambridge, Massachusetts, United States of America; 4 Khaled bin Sultan Living Oceans Foundation, Landover, Maryland, United States of America; University of Hull, United Kingdom

## Abstract

**Background:**

Fishery management has historically been an inexact and reactionary discipline, often taking action only after a critical stock suffers overfishing or collapse. The invertebrate ornamental fishery in the State of Florida, with increasing catches over a more diverse array of species, is poised for collapse. Current management is static and the lack of an adaptive strategy will not allow for adequate responses associated with managing this multi-species fishery. The last decade has seen aquarium hobbyists shift their display preference from fish-only tanks to miniature reef ecosystems that include many invertebrate species, creating increased demand without proper oversight. The once small ornamental fishery has become an invertebrate-dominated major industry supplying five continents.

**Methodology/Principal Findings:**

Here, we analyzed the Florida Marine Life Fishery (FLML) landing data from 1994 to 2007 for all invertebrate species. The data were organized to reflect both ecosystem purpose (in the wild) and ecosystem services (commodities) for each reported species to address the following question: Are ornamental invertebrates being exploited for their fundamental ecosystem services and economic value at the expense of reef resilience? We found that 9 million individuals were collected in 2007, 6 million of which were grazers.

**Conclusions/Significance:**

The number of grazers now exceeds, by two-fold, the number of specimens collected for curio and ornamental purposes altogether, representing a major categorical shift. In general, landings have increased 10-fold since 1994, though the number of licenses has been dramatically reduced. Thus, despite current management strategies, the FLML Fishery appears to be crawling to collapse.

## Introduction

The global trade of live tropical reef organisms is a multi-billion dollar industry [1], [2], [3], fueled by the demand for live food fish markets, traditional medicines, pharmaceutical and research industries, and the aquarium, jewelry and curio trades. The collection of live fish and invertebrates for home and public aquaria expanded from a small cottage industry in Sri Lanka in the 1930s, extending through the 1950s in the Philippines and Hawaii. By the 1980s, the live organism trade had grown into a major industry with collectors throughout Southeast Asia, the Pacific Islands, the Red Sea, Brazil, Hawaii, Florida and the Caribbean and recently, European species have entered the trade [4]. Currently, over 45 countries supply an estimated 30 million reef fishes annually in a global trade that includes over 1400 species. In addition, the last decade has seen aquarium hobbyists shift their display preference from fish-only tanks to miniature reef ecosystems that include many invertebrate species. Today's tanks are scale models of wild reefs where the dominant biomass includes reef-building corals assembled around a framework of “live rock” [5]. This demand for coral has led to an explosion in the live coral trade with annual increases of 10–50% since 1987. In 2005, over 1.5 million live corals and 1.5 million kg of live rock were traded [6]. The high value of this trade, estimated globally at $USD 200–330 million annually, is one factor fueling growth of this industry, but the growth continues without full consideration of the impacts to coral reefs[7].

The potential environmental and biological impacts of the ornamental fishery are widespread and long-lasting. In addition to biodiversity loss due to overfishing and selective removal of rare species [7], [8], widespread use of cyanide in collection of reef fish has caused considerable coral reef degradation [7], [9]. While these threats have long been recognized, to date, conservation efforts have concentrated on Pacific ornamental fisheries, with almost no attention paid to the Caribbean catch. Disturbingly, Caribbean reefs are among the most degraded worldwide [10], and collection of species on Caribbean reefs may lead to a further decline of reef health, and perhaps accelerate phase shift transitions from coral dominated to algal dominated reefs. This process has been termed ‘the slippery slope to slime’ and reversal has yet to be demonstrated on a large geographic scale [11], [12]. Shifts in ecosystem balance and overall changes in resilience are of a primary concern for managers (see [13] for review).

All species collected in ornamental fisheries have inherent value to the immediate location from which they were collected (ecosystem process, [14]) as well as a functional value to the consumer (ecosystem service, [14], [15]). Ecosystem processes are the large scale organization of the biotic and abiotic interactions of the natural world [14], [16]. For the marine invertebrates focused on in this study, ecosystem processes encapsulate the biological role of the organism in their natural habitat, and include roles as bioturbators, filterers, habitat providers, scavengers, predators, or grazers. Ecosystem services are a subset of the ecosystem processes that have value to humans in that they “sustain and fufill human life” [17], [18]. Organisms collected for the aquarium trade thus meet the definition of an ecosystem service, since ornamental resources [14] meet human needs through “aesthetic beauty and intellectual stimulation that lift the human spirit” [18]. Yet, the term ecosystem service is not always used to differentiate between commodities that are goods versus services [16]. This distinction is important because living organisms may be collected to meet a variety of objectives that may or may not align with the original ecosystem process, or natural function. For instance, if an organism is selected for splendor within a tank, this purely aesthetic use is disconnected from its original ecosystem service. For example, the shrimp *Hymenocera elegans* contributes a vital ecosystem function in the wild by regulating the number of predatory sea stars on the reef, but in captivity this species is traded purely for its ornamental beauty. Other organisms traded only for aesthetics include species collected for the curio and jewelry trade.

**Figure 1:**
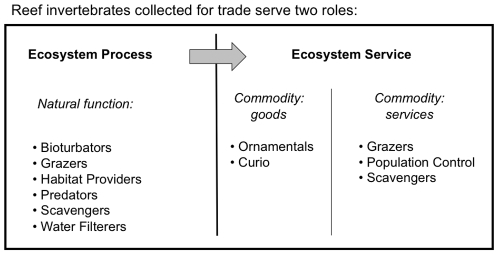
Ecosystems Processes and Ecosystems Services as they relate to the marine ornamental invertebrate trade.

In other cases, an organism may be selected to provide a role within a home aquarium that is equivalent to that of the organism's original ecosystem process. As miniature reef ecosystems are re-created in home aquariums, collection and sale of organisms that provide an ecosystem service analogous to their ecosystem process have increased [19]. For instance, the peppermint shrimp *Lysmata boggessi*, a scavenger in rocky shorelines or sea grass beds, is highly sought after for its biological control of the pest anemone *Aiptasia pallida* in aquariums [20]. Understanding the roles of organisms in their natural, versus their captive, environments is critical to gauge the future demand for species, as well as the impact of their removal from the natural environment. To facilitate the discussion of the potentially different roles that organisms have in home aquariums, we demarcated ecosystem services as commodities that represent services, such as those organisms that are placed within a home aquarium to provide a similar function to what they provided in nature (Fig. 1). This contrasts with commodities that represent goods, where organisms have a different, non-functional role in the home aquarium that does not match the natural function of their ecosystem process (Fig. 1).

**Figure 2:**
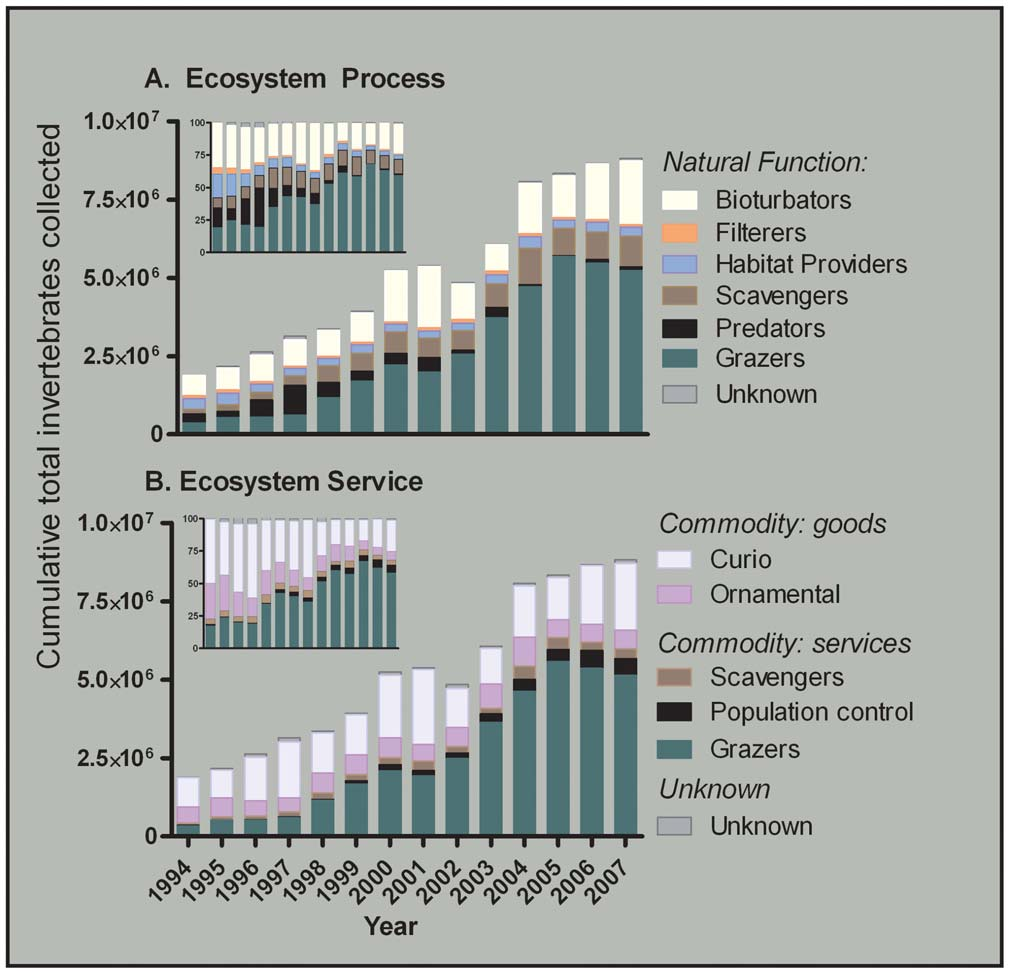
Florida Marine Life (FLML) invertebrates collected (1994-2007). Categorized by (**A**) Ecosystem Process in their natural reef, mudflat, or seagrass habitat, and (**B**) Ecosystem Service, with commodities as either goods or services depending on their purpose in the economic sector, including curio collectables and live aquarium animals. Inlays represent the percent total catch.

The U.S. State of Florida provides an excellent example of a multi-species ornamental fishery that supports the marine aquarium and curio trade. Currently, landings of over 9 million individual animals, comprised of over 600 fish, invertebrate and plant species, are reported yearly. Laudably, Florida maintains one of the most extensive data set of any ornamental fishery worldwide, recording catch landings for the ornamental and curio markets since 1994 (Fig. 2). Florida is the largest ornamental fishery in the U.S in terms of species diversity and landing numbers [21], and on a global scale, is third only to Indonesia and the Philippines [6]. The management of this multi-species fishery is primarily based on input controls [22], with limits presently capped at 168 commercial “marine life” licenses (locally referred to as “endorsements”). This license type allows collectors to capture any marine life species, with the exception of stony corals, live rock, sea fans and certain threatened fishes. Non-specific licensing has led to an asymmetry between collectors because each is legally allowed to collect all of these 600+ species, but different collectors target different taxa disproportionately. For all taxa, there are few, if any, limits on season, size, or daily catch. Such asymmetry presents management challenges that result in an inability to control the total catch effort of any given species. In addition to a commercial fishery, Florida allows recreational saltwater fishing license holders to collect a small bag limit. To our knowledge, there is currently no accounting method for recreational catches. Any additional collection from the recreational fishery will likely mirror the commercial catch, and overall will add to the numbers of individuals removed from the wild beyond those officially reported.

One difficulty with fisheries managed solely with input control is that they often lead to overfishing and stock declines [23]. Output controls exist for only a few ornamental species and are primarily volumetric based “bag” limits, but there are no output controls in the Florida fishery [24] such as catch quotas or total allowable catch. Fortunately, the FL ornamental invertebrate fishery has virtually no destructive bycatch, as most collected animals are gathered by hand to ensure quality. Cyanide is not used within this specific fishery. Still, output measures determined through fishery-dependent data, such as effort, are difficult or impossible to assess because not all licenses report landings on all species, and not all fishermen target the same species. Furthermore, the details of effort, (i.e. quantity of gear and time) are not reported or measured. Thus, while CPUE (Catch Per Unit Effort) is commonly used for fishery management, it cannot be applied in this case.

In this paper, we assess the trends in species composition of the FL ornamental invertebrate fishery between the years of 1994 and 2007. To our knowledge, this is the first analysis of this type for this fishery. In addition to the overall trends, the ecosystem service of the collected animals is of particular interest, and we assess the composition of the catch in terms of commodities that are goods versus services. We compare the changes in this fishery to the number of licenses and evaluate effectiveness of license restructuring measures as indicated by changes in landings within this fishery.

## Methods

Florida law [25] dictates that all marine fisheries products caught and sold in the state are reported in the Marine Fisheries Trip Ticket Program. On the trip ticket, wholesale dealers are required to report collector license numbers, the location of the catch, quantity, size (if applicable) and the value of each transaction by species [26]. The Florida Fish and Wildlife Conservation Commission is responsible for collecting these data, while the database is maintained by the Fish and Wildlife Research Institute (FWRI). For this study, FWRI provided anonymized trip ticket data from 1994 to 2007. Anonymizing the data is a measure to protect the identity of the fisherman, as required by law. FWRI only relinquished data for species where landings were reported by 3 or more license holders, which prevents determination of the performance of any individual fishermen. Thus, no data are available for very sparsely collected species, which are sparsely collected due to their rarity or unpopularity or atypical use, such as for research.

To determine the diversity, abundance and potential environmental impact of species collected, the data were organized by both ecological service and economic goods for each reported species. Specifically, each species in each year from 1994–2007 was binned by their respective ecosystem process (in the wild) and were re-classified to also reflect their ecosystem services (commodities as goods or services). Details of categorization are described for each species in Table S1. In general, all species codes (n = 113 codes; 72,569,069 individuals) were assigned into one of the following ecosystem process groups: bioturbators (n = 10 species codes; 17,201,409 individuals), grazers (n = 29 codes; 36,492,369 individuals), habitat providers (n = 12 codes; 3,998,106 individuals), predators (including corallivores and spongivores, total n = 19 codes; 4,427,687 individuals), scavengers (including cleaners, total n = 17 codes; 8,563,835 individuals), and water filterers (n = 21 codes; 1,211,260 individuals). Unclear landing codes (e.g. “other invertebrates”) where the ecosystem process could not be determined were lumped as its own code (unknown) for assessment (n = 5 codes; 674,402 individuals). For the ecosystem services, species were binned into one of five groups: the distinction of commodities that provide services, such as aquarium-based biological control (n = 30 codes; 41,721,081 individuals) distinguished by trophic category [grazers (n = 17 codes; 3,5650,075 individuals), scavengers (n = 12 codes; 3,211,699 individuals) or population control (n = 1 code; 2,859,307 individuals)], or distinction of ornamental (aesthetic) goods in a home aquarium (n = 57 codes; 8,510,366 individuals), or for use as curio goods (n = 9 codes; 21,275,599 individuals), or unknown (n = 17 codes; 1,062,023 individuals). Species that were reported as captive-reared, live rock, and live sand were omitted.

To analyze these fishery data, the trend, directionality, and correlation between subsequent years was assessed. For trend and directionality, the yearly landings data within each grouping were log-transformed and regressed against year. To determine patterns between years, the data were assessed by determining autocorrelation between subsequent yearly landings. A Ljung-Box Q statistic (JMP 8.0), which is a time series analysis, was calculated using a 1-year lag phase, and a significant value for this statistic indicates highly autocorreclated data [27]. Finally, the percent change in landings between years was determined, and the data presented as an average and 95% confidence interval. To more closely examine how species composition changed over the course of the time series, species (denoted by landing code) were ranked by abundance within each year. These statistics were compiled side-by-side and assigned a qualitative interpretation category based on these quantitative results (Table 1).

**Table 1:** Time series analysis of the Florida ornamental invertebrate fishery between 1994 and 2007.

	Number per year		Log(Number per year)		Aurocorrelation	Percent Change	Interpretation
	2007	b	r^2^	F_1,12_	Ljung-Box Q	per year	
***Grand Total***	*8,824,165*	*579,767*	*0.96*	*329.9* ***	*10.5* ***	*13.3±7.1*	*S ↑ Con*
**Ecosystem Process**							
Bioturbators	2,035,566	89,320	0.60	20.8***	4.5*	14.4±19.9	S ↑ Var
Grazers	5,232,479	45,6316	0.95	237.7***	11***	25.6±15.3	S ↑ Con
Habitat Providers	292,201	−366	−0.08	0.003	1.7	0.5±10.2	NS Var
Predators	114,045	−33,767	0.38	9.1*	5.2*	35.9±69.5	S ↓ Var
Scavengers	985,328	68,546	0.84	69.1***	8.8**	18.7±14.9	S ↑ Con
Water Filterers	73,147	−302	−0.07	0.07	6.7**	−1.2±9.1	NS Var
Unknown	91,399	20	−0.04	0.44	0.01	190.1±256.3	NS Var
**Ecosystem Service**							
*Commodities: service*							
Biological Control	5,989,721	513,148	0.95	258.2***	11.0***	24.6±13.3	S ↑ Con
Grazers	5,138,913	449,110	0.95	237.7***	10.9***	26.4±15.6	S ↑ Con
Population Control	534,496	43,740	0.88	97.5***	11.9***	46.3±56.0	S ↑ Var
Scavengers	316,312	20,297	0.70	31.4***	5.8**	18.3±24.2	S ↑ Var
*Commodities: goods*							
Curio	2,113,229	53,669	0.23	4.9*	1.4	11.4±17.9	NS Var
Ornamental	600,454	11,027	0.09	2.3	1.7	3.2±11.3	NS Var
Unknown	120,761	1,922	0.02	1.3	0.1	87.1±106.8	NS Var

The total species are categorized either by ecosystem process or by ecosystem service. The total number of individuals landed in 2007, and the slope of linear regression (b) is provided. Linear regressions were examined for statistical significance using log transformed data, and the Ljung-Box Q score as a measure of aurocorrelation between subsequent years. Asterisks denote level of statistical significance;

* p<0.05;

** p<0.01;

*** p<0.001.

The percent change per year is recorded as a mean ±95% confidence interval. The interpretation of these results is noted as if the change is significant or not (S or NS), if the trend is increasing or decreasing (only when significant), and if there is high variation between yearly landings (Var), or if the trends are consistent (Con).

Comparison between years focused on the oldest and most recent years, 1994 and 2007. The top 15 most heavily collected species for both years were reported along with their purpose (ecosystem service with commodity as a good or service) as well as the cumulative percent of the overall number of individuals collected for that year. Finally, we grouped the top 15 species (the most heavily collected) by taxonomic phyla (Echinodermata, Gastropoda, Arthropoda (Crustacea), and Cnidaria) and plotted the number of both licenses and landings from 1994–2007.

## Results

**Figure 3:**
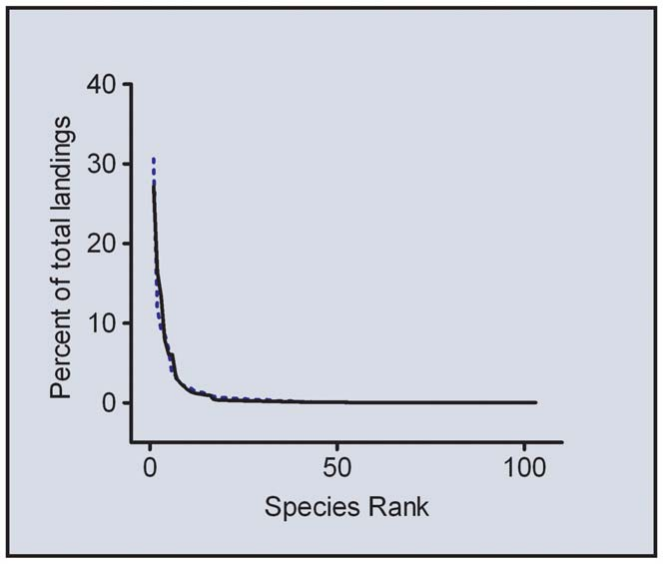
Rank abundance for species collected in the Florida marine life fishery as reported in the Trip Ticket Database. Dashed lines represent 1994 landings, Soild Lines are 2007 landings.

We found a dramatic increase in total fishery landings over the past 13 years (Fig. 2). Each year, 13.3±7.1% more individuals were landed, which is the equivalent of over 500,000 individuals yearly (Table 1). While more individuals were landed, the beginning and end years of this data set are similar in that the top 15 species account for the vast majority of landings in any given year. In 2007, 103 invertebrate landing codes corresponding to individual species or species clusters were collected in the Florida fishery, and the top 15 of these represent 92% of all reported landings (Fig. 3, Table 2). This trend is similar to that observed in 1994, in which 88 species codes were landed, and the top 15 of those were responsible for 88% of the total catch (Fig. 3, Table 2). The composition of the top 15 has changed between 1994 and 2007, and only 9 species codes are common to both lists (Table 2). The change in species composition is a result of a shift from the utilization of ornamental to biological control species in home aquariums (Fig. 2, Table 2). In 1994, six of the top 15 species caught had an ecosystem service demarcation of commodity: services, while 9 of the top 15 in 2007 provided this function.

**Table 2:** The 15 top ranked species (or species landing code) by the cumulative number of individuals landed for 1994 and 2007.

	1994				
*Rank*	*Species Description*	*Likely Scientific Identity*	*Landings*	*Cum %*	*Ecosystem Service*
1	**SAND DOLLARS**	*Mellita isometra*	578,474	30.6	Curio
2	**ANEMONE, GIANT CARIBBEAN**	*Lysmata* spp.	227,328	42.7	Ornamental
3	**SNAIL, TURBONELLA**	*Turbo* spp	173,905	51.9	Grazers
4	**SEA STAR, OTHER**	*unknown* Echinoderm	163,516	60.5	Curio
5	STARFISH, COMMON	*Asterias forbesi*	127,027	67.3	Curio
6	FILECLAM, FLAME SCALLOP	*Lima scabra*	65,155	70.7	Ornamental
7	SEA BISCUIT	*Clypeaster* sp.	64,431	74.1	Curio
8	**CRAB, THINSTRIPE HERMIT**	*Clibanarius vittatus*	45,407	76.5	Ornamental
9	**SNAIL, OTHER**	unknown grazers (*Cerithium* sp.)	41,627	78.7	Grazers
10	ANEMONE, RINGED (CURLIQUE)	*Bartholomea annulata*	35,098	80.6	Ornamental
11	**SNAIL, TOP**	*Cittarium pica*	34,408	82.4	Grazers
12	**SNAIL, STAR**	*Lithopoma americanum*	27,548	83.9	Grazers
13	**SHRIMP, PEPPERMINT**	*Lysmata* spp.	25,167	85.2	Pop control
14	ANEMONE, BANDED ( = ROCK)	*Epicystis crucifer*	24,426	86.5	Ornamental
15	URCHIN, PINCUSHION	*Lytechinus variegatus*	20,900	87.6	Grazers
	2007				
1	CRAB, BLUE-LEGGED HERMIT	*Clibanarius tricolor*	2,396,012	27.2	Grazers
2	SAND DOLLAR, 5-HOLED KEYHOLE	*Mellita tenuis*	1,453,111	43.6	Curio
3	**SNAIL, STAR**	*Lithopoma americanum*	1,184,528	57	Grazers
4	**SNAIL, TURBONELLA**	*Turbo* spp	697,639	64.9	Grazers
5	**SAND DOLLARS**	*Mellita isometra*	537,232	71	Curio
6	**SHRIMP, PEPPERMINT**	*Lysmata* spp.	534,496	77.1	Pop control
7	SNAIL, TURBO (CORALLINE RED ALGAE)	*Lithopoma* spp.	271,586	80.2	Grazers
8	**SNAIL, OTHER**	unknown grazers (*Cerithium* sp.)	227,391	82.7	Grazers
9	SNAIL, BRUISED NASSA	*Nassarius vibex*	183,550	84.8	Scavenger
10	**SNAIL, TOP**	*Cittarium pica*	147,432	86.5	Grazers
11	**CRAB, THINSTRIPE HERMIT**	*Clibanarius vittatus*	120,255	87.9	Ornamental
12	CORAL, MUSHROOM (CORALLIMORPHS)	*Ricordia florida*	106,425	89.1	Ornamental
13	CRAB, GREEN REEF (CLINGING)	*Mithraculus sculptus*	98,459	90.2	Grazers
14	**ANEMONE, GIANT CARIBBEAN**	*Condylactis gigantea*	91,737	91.2	Ornamental
15	**SEA STAR, OTHER**	unknown Echinoderm	86,222	92.2	Curio

Cumulative percent is calculated utilizing the total number of individuals landed in each year. Species in bold appear on both lists.

In consideration of the ecosystem service that the collected species provide, the most rapid increase was with species that were traded as commodities for their services (statistical details shown in Table 1). The commodities as goods (curio and ornamental) and unknown species demonstrated no autocorrelation between years; the percent increase per year was not significantly different from zero (statistical details shown in Table 1). The curio species did exhibit a significant linear regression of year vs. number landed (statistical details shown in Table 1), but if the p-value is protected for multiple comparisons (6 total ecosystem service categories, protected p = 0.008), then this result would be spurious. Grazers constituted the majority of the service-providing species, and were the only group to demonstrate a statistically significant increase per year (26.4±15.6%, Fig. 2A, statistical details shown in Table 1).

In the 1990s, charismatic invertebrates represented nearly 80% of the 1.8 million individuals collected (Fig. 1B, inlay). In 2007, however, charismatic collections represented 30% of the nearly 9 million specimens. While their rank abundance has decreased, the total number of charismatic specimens collected per year has increased within the 13 year dataset, and exceeds the total number of individuals collected in the first data year. The number of ornamentals collected has remained relatively constant since 1994, while the number of curio specimens collected have nearly doubled (Fig. 2B, Table 2). The remaining 6 million individuals collected in 2007 were predominantly grazers, and the number of grazers now exceeds, by two-fold, the number of specimens collected for curio and ornamental purposes altogether, representing a major categorical shift (Fig. 2B).

**Figure 4:**
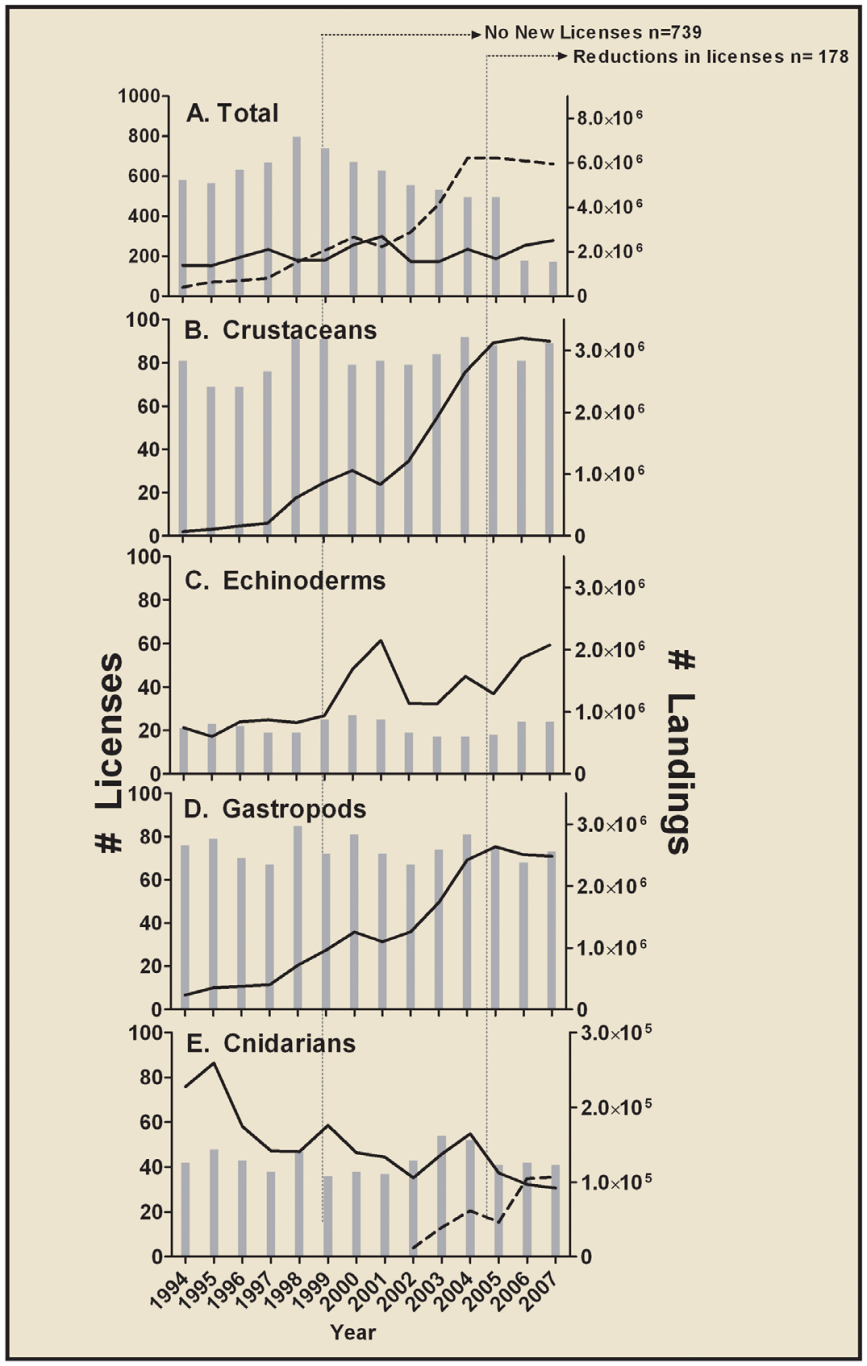
Florida marine life fishery trends from 1994 to 2007, number of licenses (bars) and landings (lines). (**A**) Total number of licenses held and landings reported in the trip ticket database for each year from 1994 to 2007. Solid line is commodity goods, broken line is commodity services. (**B–E**) Number of active licenses and landings for the top 15 most heavily collected species grouped by taxonomic category. Categories (**B**) and (**D**) are strictly traded for their services, categories (**C**) and (**E**) are traded as goods. For (**E**), solid line represents *Condylactis gigantea*, broken line represents corallimorphs, primarily *Ricordea florida*.

Even though the number of active licenses reporting landings has remained relatively stable over time for the most heavily collected species, fishing pressure has increased in almost every species over the past decade (Fig. 4). This upwards trend is independent of license number; in reality, only a few licenses represent the majority of the landings (Fig. 4A). For example, most of the 9 million specimens landed in 2007 were collected by only ∼40% of active FLML licenses (Table 2). Florida's efforts to reduce the number of available licenses in the fishery (a moratorium on new licenses, and a reduction in current licenses; Fig. 4A) have succeeded only in removing inactive or part-time fisherman. Thus, the majority of ornamental fishing effort has remained unaffected by all of Florida's regulatory action to date, and the number of landings continues to grow unchecked (Fig. 1; Fig. 4A, Table 1). Furthermore, the shift from fish-only to invertebrate-dominated ecosystem aquaria rapidly altered the suite of species being collected and the current regulations for this multi-species fishery are not sufficient to prevent overfishing.

The decline in landings of the anemone *Condylactis gigantea* (coded in Table 2) represents an interesting case study. Whereas landings of other invertebrates are steadily increasing in number despite license reductions, *C. gigantea* have experienced a steady decline in reported landings (Fig. 4E). In 1994, 227,238 individuals were landed, while in 2007, this number was only 91,737 (Table 2). Rather than reflecting restrictions on effort or a decline in popularity (it is on both the 1994 and 2007 top 15 species list, Table 2) the decrease in landings instead reflect its increasing rarity, as this species is widely considered to be overfished [28], [29]. Even though the overall trend for charismatic invertebrates (ornamental and curio) is to represent a smaller percent of the total landings (80% in 1994 compared to 30% in 2007), the overall number of these individuals landed in the fishery doubled from 1.4 to 3 million in this time span. Thus any reduction in landings may signal larger problems in this invertebrate fishery as the decrease is likely a result of a lower abundance rather than a lessening in demand. Indeed, the rapid increase in landings and number of fisherman reporting catches of *Ricordea florida* corallimorphs (coded in Table 2; Figure 4) is likely another indicator of the complexity of managing this multi-species fishery. This landing code was not present in the database for 1994 (landing code added in April 2002), and by 2007, was the 12th most popular species collected (106,425 individuals, Table 2). When a species becomes popular, the number of licenses reporting landings can dramatically increase despite the reduction in the total number of licenses within the fishery.

## Discussion

Despite the dramatic changes in the Florida invertebrate ornamental fishery, there have been no *in situ* studies assessing the impacts of invertebrate herbivore removal beyond *Diadema antillarum* urchins [e.g. 30], [31], which are not a part of the ornamental fishery. For most invertebrates, the reproductive age, growth rate, population density/distribution, and population connectivity remain elusive. Furthermore, it remains unknown whether cryptic populations or species exist, or whether these invertebrates have the ability to withstand rapid changes in fishery pressure. Despite a general trend of a minority of licenses reporting landings on any given species, frequently fished invertebrates have experienced 10-fold increases in landings since data have become available. Without these invertebrates, are important reef ecosystems crawling to their eventual collapse?

The time series analysis of the data for the years 1994 to 2007 demonstrated a shift in the ecosystem services provided by invertebrates collected for the home aquarium and curio trades. Overall, while there is an increase in all categories of species collected, those species that provide a service in the home aquarium are being collected at an increasingly rapid pace. Specifically, grazers are being collected the most because they serve a key ecological role in home aquaria by controlling algal growth. However, they perform an analogous ecosystem process in the wild and thus their removal may greatly impact their natal reef. A growing body of evidence supports the idea that removing grazers decreases the resilience of a reef ecosystem, thereby reducing its ability to withstand a phase-shift from a coral to an algal-dominated state as well as decreasing the potential for subsequent phase-shift reversals [32].

FLML currently operates as a demand regulated fishery, meaning that landings increase with demand. Given the stark outlook for the global economy at the present time, and given that marine home aquaria are “luxury” expenditures, growth in ornamental fisheries is expected to slow or decrease. While industry demand is slow, a limited window of opportunity is open where management policies can change without immediate disruption of economic livelihood. During this time, managers could consider switching from their current strategy to a mixed-adaptive strategy. The new strategy would consist of single species management for the top 15 species, which represent a majority of the individuals landed, with the remainder managed within a multi-species plan. Currently for the top 15 species, there are no size restrictions, spawning stock considerations, seasonal closures, or other ecological considerations. Furthermore, taxonomic loopholes exist that allow unrestricted fishing of complimentary regulated species. Thus, it may be most adaptive to manage this fishery in terms of species complexes as opposed to single species. Over time, the management strategy could adapt to accommodate the top 15 species (or species complexes) as those that are rarely collected become more commonly collected in the future.

A further overlooked nuance of limited entry fisheries is that license transfers can dramatically impact fishing pressure for a given species. Because these are multi-species licenses, different species or species clusters may be collected by the differing license owners. Unlike highly mobile fish, slow or sessile invertebrates are exceptionally vulnerable to these license transfers because newcomers to the industry are less skilled than seasoned fisherman; thus, they are likely to target slow-moving invertebrate species. Given that on average, less than half of the licenses report landings for a given species, managers should also consider further reductions in the number of licenses within the fishery to ensure that license transfer do not rapidly alter catch composition. While the cost of entry into the fishery is currently high (30,000 USD), price is set by the open market and fluctuates with demand and availability. Thus, license cost in this fishery is not a means to limit license transfers. Changes in license ownership have the potential to dramatically alter the catch composition, landing numbers, or fishing location, which has important consequences since most of the species in Florida are collected in the Florida Keys National Marine Sanctuary (FKNMS).

There is current legislation being considered that would eliminate the trade in non-native wildlife (i.e. H.R. 669, Nonnative Wildlife Invasion Prevention Act). If this or a similar measure are adopted, they could abolish or limit the import of species from anywhere outside of the United States. This would, in turn, dramatically increase the pressure on Florida to exclusively provide ornamental species for U.S. home aquaria. To date, the data collected on imported aquarium ornamentals is sparse and only includes abundance and country of origin [33]. While there is taxonomic and life history stage data available at the time of import, there is no database information available for fisheries use. This data gap on international ornamental imports prevents predictable forcasting; in other words, the inevitable increased pressure on Florida fisheries is not quantifiable at this time.

Fishery management has historically been an inexact and reactionary discipline, often taking action only after a critical stock suffers overfishing or collapse. Management challenges arise from ecosystem complexity and a paucity of baseline data, resources and support. Often, small fisheries grow beyond their intended capacity. In Florida, the once small ornamental fishery is now an invertebrate-dominated industry supplying five continents. As a result, the FLML may be positioned for collapse. However, fishermen in the FKNMS who have collected specimens for the aquarium trade for more than 4 decades strongly advocate for stricter licensing, catch-limits on key species, and environmental monitoring programs. Such citizen-driven management concern is encouraging, but it also highlights the severity of ecosystem collapse. After all, would this fishing industry subject itself to such restrictions if resources were plentiful? This fishing community is admirably and desperately trying to avoid the classic “tragedy of the commons” in order to preserve their livelihood.

If much-needed regulations are implemented in some regions, and other regions suffer from massive declines, which ecosystem will be called upon to supply the invertebrate trade, and at what ecosystem cost? Sustainable fisheries are often called for, but hardly defined. Considering ornamental invertebrates outside of the food industry is vital whether they swim or crawl, as collapse may be on the horizon for many of these overlooked species.

## Supporting Information

Table S1 Landing codes and binning data for the FLML fishery, with landing codes as of July 2009.
(0.11 MB DOC)
