# Supplementary material for: Crawling to Collapse: Ecologically Unsound Ornamental Invertebrate Fisheries
Source: PLoS One. 2009 Dec 22;4(12):e8413. doi: 10.1371/journal.pone.0008413 (PMC2793429; doi:10.1371/journal.pone.0008413)
Supplement: Table S1 — Landing codes and binning data for the FLML fishery, with landing codes as of July 2009. (0.11 MB DOC) [file pone.0008413.s001.doc]

| **Code** | **Previous Restriction (10/30/2007)** | **Current Restriction** | **Description** | **Ecosystem Services** | **Ecosystem function** |
| --- | --- | --- | --- | --- | --- |
| 701 | M | M | Anemone, sun (=carpet) | Ornamental | Habitat Providers |
| 702 | M | M | Anemone, white/brown colonial (Palythoa) | Ornamental | Habitat Providers |
| 703 | M | M | Anemone, giant Caribbean (=Condylactus) | Ornamental | Habitat Providers |
| 704 | M | M | Anemone, sun zoanthid (Palythoa) | Ornamental | Habitat Providers |
| 705 | M | M | Anemone, ringed (=curlique) | Ornamental | Habitat Providers |
| 706 | M | M | Anemone, green colonial (Zoanthus) | Ornamental | Habitat Providers |
| 707 | M | M | Anemone, banded (=rock) | Ornamental | Habitat Providers |
| 708 | M | M | Anemone, other | Ornamental | Habitat Providers |
| 709 | M | M | Crab, yellowline arrow | Biological Control | Grazers |
| 710 | * | M | Crab, hermit, other | Biological Control | Scavengers |
| 711 | M | M | Crab, furcate spider (=decorator) | Ornamental | Grazers |
| 712 | -- | M | Crab, green clinging or reef (emerald) | Biological Control | Grazers |
| 713 | M | M | Crab, false (=frilly) arrow | Biological Control | Grazers |
| 714 | * | M | Crab, red banded hermit | Biological Control | Scavengers |
| 715 | M | M | Crab, thinstripe (=green-striped) hermit | Ornamental | Scavengers |
| 716 | -- | M | Crab, red-legged (scarlet) hermit | Biological Control | Grazers |
| 717 | M | M | Crab, white operculate (polkadot) | Ornamental | Scavengers |
| 718 | M | M | Crab, blue-legged hermit | Biological Control | Grazers |
| 719 | -- | -- | Crab, horseshoe | Biological Control | Scavengers |
| 720 | * | M | Crab, stareye hermit | Biological Control | Scavengers |
| 721 | M | M | Crab, spotted porcelain | Ornamental | Water Filterers |
| 722 | * | M | Crab, giant hermit | Biological Control | Scavengers |
| 723 | M | M | Crab, nimble spray (=lightfoot, urchin) | Biological Control | Grazers |
| 724 | -- | -- | Crab, land hermit | Not included | Not included |
| 725 | -- | -- | Crab, other (non-ML)*** | Biological Control | Scavengers |
| 726 | * | M | Crab, white speckled hermit | Biological Control | Scavengers |
| 727 | M | M | Gorgonian, red | Ornamental | Habitat Providers |
| 729 | M | M | Gorgonian, sea blades (=purple) | Ornamental | Habitat Providers |
| 731 | M | M | Gorgonian, other | Ornamental | Habitat Providers |
| 732 | M | M | Siphonophores/Hydroids | Unknown | Water Filterers |
| 733 | M | M | Jellyfish, upside-down | Ornamental | Water Filterers |
| 734 | -- | -- | Jellyfish, other (non-ML)*** | Ornamental | Water Filterers |
| 735 | R | R | Lobster, Caribbean spiny (=rock) | Ornamental | Scavengers |
| 736 | -- | -- | Lobster, spotted | Ornamental | Scavengers |
| 737 | -- | -- | Lobster, Spanish slipper (=shovelnose) | Ornamental | Scavengers |
| 739 | M | M | Nudibranch, lettuce sea slug (=fancy) | Biological Control | Grazers |
| 740 | M | M | Nudibranch, FL regal doris (=Greek godd | Ornamental | Spongivores |
| 741 | M | M | Nudibranch, other | Unknown | Grazers |
| 742 | M | M | Sea Hare | Biological Control | Bioturbators |
| 743 | -- | -- | Octopus, common | Ornamental | Predators |
| 744 | M | M | Octopus, Caribbean reef | Ornamental | Predators |
| 745 | M | M | Octopus, Atlantic pygmy (=dwarf) | Ornamental | Predators |
| 746 | M | M | Octopus, white-spotted | Ornamental | Predators |
| 747 | -- | -- | Oyster, Atlantic thorny- (=spiny) | Ornamental | Water Filterers |
| 757 | -- | -- | Sand dollar, other | Curio | Bioturbators |
| 758 | -- | -- | Sea biscuit, other | Curio | Bioturbators |
| 759 | M | M | Fileclam, rough (=flame scallop) | Ornamental | Water Filterers |
| 761 | M | M | Fileclam, spiny (=white flame scallop) | Ornamental | Water Filterers |
| 763 | M | M | Sea cucumber, Florida | Biological Control | Bioturbators |
| 764 | M | M | Sea cucumber, other | Biological Control | Bioturbators |
| 765 | M | M | Shrimp, spotted cleaner (=anemone) | Ornamental | Cleaners |
| 766 | -- | -- | Shrimp, mantis | Ornamental | Bioturbators |
| 767 | M | M | Shrimp, banded coral (=barber pole) | Ornamental | Cleaners |
| 768 | R | -- | Shrimp, rock | Unknown | Unknown |
| 769 | M | M | Shrimp, peppermint (=veined) | Biological Control | Scavengers |
| 770 | * | M | Shrimp, Pederson's cleaner | Ornamental | Cleaners |
| 771 | M | M | Shrimp, pistol or snapping | Ornamental | Bioturbators |
| 773 | M | M | Shrimp, cleaner (=scarlet cleaner) | Ornamental | Cleaners |
| 774 | -- | -- | Shrimp, other (non-ML)*** | Not included | Not included |
| 775 | -- | -- | Cowrie, measled | Ornamental | Grazers |
| 776 | -- | -- | Cowrie, Atlantic deer | Ornamental | Grazers |
| 777 | -- | -- | Snail, flamingo tongue | Ornamental | Corallivores |
| 778 | -- | -- | Snail, sharkeye (=Atlantic moon) | Unknown | Predators |
| 779 | -- | -- | Conch, Florida horse | Curio | Predators |
| 780 | * | -- | Snail, cerith | Biological Control | Grazers |
| 781 | -- | -- | Snail, tulip (=true tulip) | Unknown | Grazers |
| 785 | -- | -- | Snail, chestnut turban | Biological Control | Grazers |
| 787 | -- | -- | Snail, other (non-ML)*** | Biological Control | Grazers |
| 788 | * | M | Sponge, other (ML only) | Ornamental | Water Filterers |
| 789 | M | M | Sponge, red ball | Ornamental | Water Filterers |
| 790 | M | M | Sponge, red finger | Ornamental | Water Filterers |
| 791 | M | M | Sponge, red tree | Ornamental | Water Filterers |
| 792 | -- | -- | Sponge, other (non-ML)*** | Ornamental | Water Filterers |
| 793 | M | M | Sea star, red spiny (=common) | Curio | Predators |
| 796 | M | M | Sea star, other | Curio | Predators |
| 797 | M | M | Basket star | Ornamental | Water Filterers |
| 798 | M | M | Sea lilies (Crinoids) | Ornamental | Water Filterers |
| 799 | M | M | Brittle star, spiny ophiocoma | Biological Control | Scavengers |
| 801 | M | M | Brittle star, red serpent | Biological Control | Scavengers |
| 802 | -- | -- |  |  |  |
| 803 | M | M | Brittle star, serpent | Biological Control | Scavengers |
| 804 | M | M | Brittle star, other | Biological Control | Scavengers |
| 806 | -- | M | Urchin, purple-spined (Arbacia) | Biological Control | Grazers |
| 807 | M | M | Urchin, pencil | Biological Control | Grazers |
| 809 | M | M | Urchin, variable or green (pincushion) | Biological Control | Grazers |
| 810 | -- | M | Urchin, other | Biological Control | Grazers |
| 811 | M | M | Urchin, rock-boring (red; Echinometra) | Biological Control | Grazers |
| 812 | -- | -- | Live rock (pounds)* | Not included | Not included |
| 827 | -- | -- | Live sand | Not included | Not included |
| 829 | -- | -- | Bryozoa | Unknown | Water Filterers |
| 830 | -- | -- | Tunicates, sea squirts | Ornamental | Water Filterers |
| 831 | M | M | Corallimorph, Ricordea | Ornamental | Habitat Providers |
| 832 | * | M | Corallimorph, Discosoma | Ornamental | Habitat Providers |
| 833 | * | M | Corallimorph, other | Ornamental | Habitat Providers |
| 834 | * | M | Crab, clinging reef (M. ruber) | Biological Control | Grazers |
| 835 | -- | -- | Crab, box (=flame) | Ornamental | Predators |
| 836 | * | M | Crab, red-ridged clinging | Biological Control | Grazers |
| 837 | -- | -- | Crab, redfinger rubble (=calico) | Ornamental | Predators |
| 838 | * | -- | Crab, clinging reef, other (non-ML)*** | Biological Control | Grazers |
| 839 | -- | -- | Clam, sunray Venus | Unknown | Unknown |
| 841 | -- | -- | Scallop, lions-paw | Ornamental | Water Filterers |
| 843 | -- | -- | Scallop, other | Ornamental | Water Filterers |
| 845 | -- | -- | Clam, angel wing | Unknown | Unknown |
| 847 | -- | -- | Clam, jewel box | Ornamental | Water Filterers |
| 849 | -- | -- | Clam, other | Ornamental | Water Filterers |
| 904 | -- | -- | Crab, fiddler | Unknown | Unknown |
| 935 | * | -- | Snail, Eastern mud | Biological Control | Scavengers |
| 937 | * | -- | Snail, Nassarius, other | Biological Control | Scavengers |
| 939 | * | -- | Snail, turbo, other (non-ML)*** | Biological Control | Grazers |
| 941 | -- | -- | Sand dollar, 5-holed keyhole | Curio | Bioturbators |
| 943 | -- | -- | Sand dollar, 6-holed keyhole | Curio | Bioturbators |
| 945 | -- | -- | Sand dollar, notched | Curio | Bioturbators |
| 946 | -- | -- | Sea biscuit, inflated | Curio | Bioturbators |
| 947 | -- | -- | Sea biscuit, flat | Curio | Bioturbators |
| 951 | -- | -- | Conch, Florida fighting | Biological Control | Grazers |
| 953 | -- | -- | Conch, milk | Biological Control | Grazers |
| 957 | -- | -- | Conch, hawkwing | Unknown | Grazers |
| 959 | -- | -- | Conch, Florida crown | Unknown | Predators |
| 960 | -- | -- | Conch, other | Unknown | Grazers |
| 961 | -- | -- | Whelk, knobbed | Curio | Predators |
| 963 | -- | -- | Whelk, lightning | Curio | Predators |
| 964 | -- | M | Snail, bruised nassa | Biological Control | Scavengers |
| 965 | -- | -- | Snail, frogsnail | Unknown | Unknown |
| 966 | -- | -- | Snail, helmet | Curio | Predators |
| 967 | -- | -- | Snail, cone | Unknown | Predators |
| 968 | -- | -- | Snail, wentletrap | Unknown | Unknown |
| 969 | -- | -- | Snail, fig | Unknown | Unknown |
| 970 | -- | -- | Snail, purple sea | Biological Control | Grazers |
| 971 | -- | -- | Snail, periwinkle | Biological Control | Grazers |
| 972 | -- | -- | Snail, marginella | Unknown | Unknown |
| 973 | -- | -- | Snail, melampus | Unknown | Unknown |
| 974 | -- | -- | Snail, Florida miter | Unknown | Unknown |
| 975 | -- | -- | Snail, rocksnail (=rock shell) | Unknown | Unknown |
| 976 | -- | -- | Snail, murex | Ornamental | Predators |
| 977 | -- | -- | Snail, moon, other | Unknown | Unknown |
| 978 | -- | -- | Snail, nerites | Ornamental | Grazers |
| 979 | -- | -- | Snail, olive | Ornamental | Bioturbators |
| 980 | -- | -- | Snail, triton, angular | Unknown | Grazers |
| 981 | -- | -- | Snail, triton, other | Unknown | Grazers |
| 982 | -- | -- | Snail, topsnail | Biological Control | Grazers |
| 983 | -- | -- | Snail, vase | Unknown | Unknown |
| 984 | M | M | Snail, star | Biological Control | Grazers |
| 985 | -- | -- | Chiton | Unknown | Grazers |
| 986 | -- | -- | Snail, tegula | Unknown | Unknown |
| 987 | -- | -- | Crab, coral spider | Ornamental | Grazers |
| 988 | -- | -- | Amphipods/Copepods | Not included | Not included |
| 989 | -- | -- | Penshell | Ornamental | Water Filterers |
| 991 | M | -- | Polychaete, horned Christmas-tree | Ornamental | Water Filterers |
| 992 | -- | -- | Polychaete, fireworm | Unknown | Predators |
| 993 | M | M | Polychaete, fanworm | Ornamental | Water Filterers |
| 994 | M | M | Polychaete, feather-duster | Ornamental | Water Filterers |
| 995 | -- | -- | Polychaete, other (non-ML)*** | Unknown | Unknown |
| 996 | -- | -- | Isopod, deepwater (Bathyonomus) | Unknown | Scavengers |
| 998 | -- | -- | Miscellaneous invertebrates | Unknown | Unknown |
| N/A | -- | -- | OTHER INVERTEBRATES | Unknown | Unknown |
| N/A | -- | -- | SNAIL, HORSE CONCH | Curio | Predators |
|  | Codes new to database, within the past 24 months, were not included in the anaylsis. | | |  |  |
|  | Landings for codes with less than 3 fisherman reporting were not provided by FWRI | | |  |  |
|  | | -- | No restriction on harvest *Not a valid trip ticket code |  | | --- | --- | --- | | M | Requires marinelife endorsement | | | | |  |  |
